# A New and Effective Method to Trace Tibetan Chicken by Amino Acid Profiling

**DOI:** 10.3390/foods12040876

**Published:** 2023-02-18

**Authors:** Mengjie Qie, Yalan Li, Xiangyu Hu, Cidan Zhaxi, Shanshan Zhao, Zixuan Zhang, Xiaoting Yang, Lu Bai, Yan Zhao

**Affiliations:** 1 Institute of Quality Standard & Testing Technology for Agro-Products, Key Laboratory of Agro-Product Quality and Safety, Chinese Academy of Agricultural Sciences, Beijing 100081, China; 2 College of Food Science and Engineering, Shandong Agricultural University, Taian 271018, China; 3 Institute of Quality Standards & Testing Technology for Agro-Product, Tibet Academy of Agricultural and Animal Husbandry Sciences, Lhasa 850032, China; 4 Institute of Food and Nutrition Development, Ministry of Agriculture, Beijing 100081, China

**Keywords:** Tibetan chicken, meat, geographical origins, amino acids, plateau, altitude

## Abstract

As a “rare bird on the plateau”, the Tibetan chicken is rich in nutrition and has high medicinal value. In order to quickly and effectively identify the source of food safety problems and to label fraud regarding this animal, it is necessary to identify the geographical traceability of the Tibetan chicken. In this study, Tibetan chicken samples from four different cities in Tibet, China were analyzed. The amino acid profiles of Tibetan chicken samples were characterized and further subjected to chemometric analyses, including orthogonal least squares discriminant analysis, hierarchical cluster analysis, and linear discriminant analysis. The original discrimination rate was 94.4%, and the cross-validation rate was 93.3%. Moreover, the correlation between amino acid concentrations and altitudes in Tibetan chicken was studied. With the increase in altitude, all amino acid contents showed a normal distribution. For the first time, amino acid profiling has been comprehensively applied to trace the origin of plateau animal food with satisfactory accuracy.

## 1. Introduction

The “roof of the world”, the Qinghai–Tibet Plateau, is located in southwest China. The plateau has a high altitude (average altitude: about 4320 m), low temperature (annual average temperature: −1.9–7.2 °C), hypobaric pressure (average pressure: 500–600 mbar), long sunshine duration (annual sunshine duration: 2600–3000 h), low rainfall (average annual rainfall: 200–300 mm), and a complicated and changeable climate [1,2,3]. Well known as the “plateau rare bird” [4], the Tibetan chicken is a native poultry breed, with a small body size, of a type this is unique to the Qinghai–Tibet Plateau. The bird is distributed at an altitude of 2200–4100 in semi-agricultural and semi-pastoral areas [5,6]. The unique living environment has resulted in the Tibetan chicken developing certain advantages, such as strong adaptability to the plateau climate, strong disease resistance, tolerance to the cold, fleshy breast and drumstick, fresh taste, special flavor, rich nutrients, and high medicinal value [4,7]. Tibetan chicken has become a popular product with a high market price and short supply, which may result in possible fraudulent labeling, due to the opportunity for large economic profits [8]. The traditional Tibetan chicken is a natural, green, and healthy ecological product, requires no special supplementary feeding, and is free-range throughout the year [4]. However, with the development of the breeding industry, Tibetan chickens are also faced with food safety problems due to multi-channel introduction, frequent circulation, lagging epidemic prevention, and sanitation technology [9]. Therefore, the origin traceability of Tibetan chickens in Tibet can help with effectively locating the origin of Tibetan chickens in the case of food safety problems, so as to realize the rapid recall of problematic products.

The methods of food traceability include indicators directly related to geographies, such as stable isotopes [8] and minerals [10], as well as fingerprint indicators of organic components, such as amino acids [11] and fatty acids [12]. Sun et al. investigated the origin assignment via the multi-element stable isotopes of lamb tissues from five different regions of China. They found that a total correct classification of 88.9% and 83.8% were obtained from the combination of the C, N, and H isotopes of lamb muscle and wool [13]. Qi et al. used a fingerprint analysis of mineral elements and machine learning to trace the origin of pork in seven regions of China, and they found that the feedforward neural network achieved a superior performance with an overall accuracy of 95.71% [14]. Moreover, Dragan et al. assessed the lambs that originated from ten different grazing areas in North Macedonia. Based on the fatty acid profiles and canonical discriminant analysis, it was shown that there was a significant linear divergence between the tissues from almost all the examined regions [15]. Regarding amino acids, Kang et al. established a method to identify the geographical origin of milk samples from four regions in China based on their amino acid profiles, obtaining a classification accuracy of 100% [16]. In addition, studies have shown that amino acid parameters have also been applied to the authentication and traceability verification in goat meat [17], goat milk [18], honey [19], and plastron-derived functional foods [20]. However, as a kind of mature detection method, the amino acid indicator has not been reported in the research field of the origin traceability of chicken. Additionally, only a few studies have indicated that the amino acid content of chicken from different origins is different [21,22].

Previously, we used stable isotope and chemometric methods to discriminate between Tibetan chickens and chickens from four other provinces in the plain region, with a cross-validation rate of 97.6% [8]. However, the highest cross-validation rate was only 61.0% when the stable isotope technique was used to distinguish the Tibetan chicken samples from the four different cities in Tibet. This fact indicates that the satisfactory traceability of Tibetan chicken cannot be achieved by the stable isotope technique. Further, based on the economic situation in Tibet, there is an urgent need for a relatively universal, easy-to-grasp, and cheap analytical technique for the purposes of accurate origin traceability, such that the amino acid profile could be tried in order to estimate the geographical origin of the Tibetan chicken. At the same time, according to the special climatic conditions in Tibet, we further analyzed the relationship between amino acid composition and the content of chicken from four different areas in Tibet. The altitude not only provides a reference, for the benefit of researchers, to plateau animal samples, but also provides new ideas for tracing the origin of plateau animal food.

In this study, Tibetan chicken samples from four regions of Tibet—Lhasa, Shannan, Linzhi, and Xigaze—were distinguished by 17 amino acids: phenylalanine (Phe), methionine (Met), lysine (Lys), threonine (Thr), leucine (Leu), isoleucine (Ile), valine (Val), aspartic acid (Asp), serine (Ser), glutamic acid (Glu), proline (Pro), glycine (Gly), alanine (Ala), histidine (His), arginine (Arg), cysteine (Cys) and tyrosine (Tyr). The correlation between amino acid composition and the altitudes in Tibetan chicken samples from these regions was investigated. The amino acid composition was found to vary in the different altitudes. In addition, the chemometric methods of the ANOVA test, linear discriminant analysis (LDA), hierarchical cluster analysis (HCA), and orthogonal partial least squares-discriminant analysis (OPLS-DA), when applied to the amino acid data. were established in order to distinguish Tibetan chickens in the four regions of Tibet. In brief, an amino acid profile combined with chemometrics was established to identify the geographical traceability of the Tibetan chicken.

## 2. Materials and Methods

### 2.1. Sample Information

**Figure 1:**
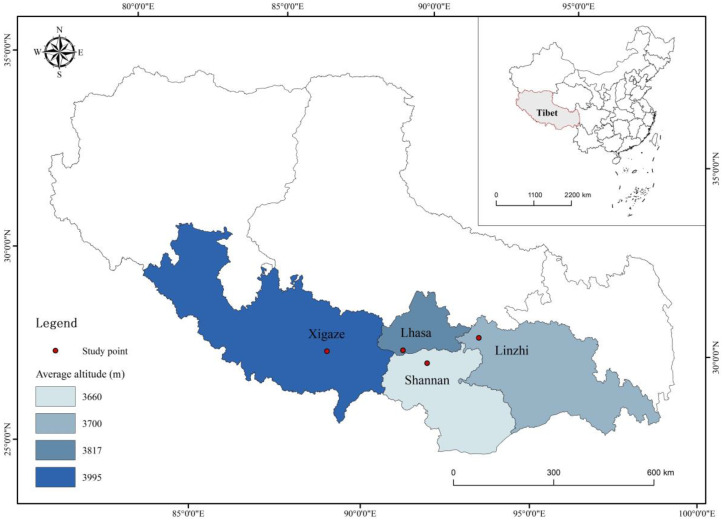
Geographical map of chicken sampling sites from Tibet.

All Tibetan chicken samples were collected from four different cities (Lhasa, Shannan, Linzhi, and Xigaze) in the Tibet Autonomous Region. Chicken samples were taken directly from local abattoirs within a period of month. The geographic location map of all the samples is shown in Figure 1. The details regarding the origin of 89 chicken samples are shown in Table 1. The chicken samples were packed in sealed polyethylene bags and stored at −20 °C.

### 2.2. Amino Acid Analysis

The pretreatment of amino acid analysis was based on GB 5009.124-2016 “Determination of Amino Acids in Food”. In brief, a 40.0 mg minced chicken breast sample was placed in a hydrolysate tube. A 15 mL 6 mol/L guarantee reagent HCl (Sinopharm Pharmaceutical Co., Ltd. Beijing, China) solution and 4 drops of chromatographic purity phenol (Merck KGaA, Darmstadt, Germany) were added to the tube. The tube was vacuum sealed and hydrolyzed in an oven at 110 °C for 22 h. It was allowed to cool, then the hydrolysate was filtered, and the volume was fixed to 50 mL with ultrapure water (A.S.Watson TM Ltd., Central and Western District, Hong Kong, China). Then, a 1.0 mL filtrate was taken for the purposes of drying under nitrogen at 50 °C. The dried residue was dissolved in 1 mL of water, dried under nitrogen, and finally evaporated to the point of dryness. Then, the residue was redissolved in a 1.0 mL guarantee reagent sodium citrate (Sinopharm Pharmaceutical Co., Ltd. Beijing, China) buffer solution (C _Na+_ = 0.2 mol/L, pH 2.2). After mixing, the supernatant was filtered through a 0.22 μm nylon membrane syringe filter (Jinteng Ltd., Tianjin, China), and transferred to a 2 mL glass vial for further analysis.

Then, a content analysis of amino acids using an automatic analysis apparatus according to the publication of Xie et al. [11] was conducted. A chromatographic analysis of the amino acids was carried out by a high-performance liquid chromatography (Waters Ltd., Milford, MA, USA) equipped with a PDA detector and 4.6 mm × 250 mm, 5 μm Universal C18 column (Kromat Corporation, Bordentown, NJ, USA). The column oven temperature was maintained at 40 °C. The detection wavelength was 360 nm. The flow rate and injection volumes were 1 mL/min and 10 μL, respectively. The mobile phase consisted of solvent A (acetonitrile, chromatographic purity, Thermo Fisher Scientific, Waltham, MA, USA) and solvent B (acetic acid-sodium acetate buffer: 0.03 mol/L sodium acetate (Sinopharm Pharmaceutical Co., Ltd. Beijing, China) solution and 0.15% trimethylamine (Merck KGaA, Darmstadt, Germany), pH 5.25 ± 0.05 with glacial acetic acid, chromatographic purity, Merck KGaA, Darmstadt, Germany). The gradient program was as follows: 18% A (0–10 min), 18–20% A (10–15 min), 20–34% A (15–30 min), 34–45% A (30–35 min), 45–55% A B (35–38 min), 55–60% A (38–42 min), and 60–18% A (42–45 min).

### 2.3. Statistical Analysis

SPSS 22.0 (International Business Machines Corporation, Armonk, NY, USA) was used for the statistical analysis. Duncan’s multiple comparisons were performed to determine the significant differences between individual regions, which were found to be significant as per ANOVA. Linear discriminant analysis was used to determine the discriminant accuracy of the chicken samples from different regions. To visually show the difference between Tibetan chicken samples in the four different cities, the OPLS-DA model and the HCA model were generated using the SIMCA 14.1 software (Umetrics, Umea, Sweden). Correlation analysis (Excel 2016, Microsoft Corporation, Washington, DC, USA) was used to explore the relationship between amino acid concentrations and altitudes in the Tibetan chicken samples. The data employed for the multivariate analysis are shown in Table S1.

## 3. Results and Discussion

### 3.1. Components and Evaluation of Amino Acids in Tibetan Chicken

The components of seventeen amino acids (Phe, Met, Lys, Thr, Leu, Ile, Val, Asp, Ser, Glu, Pro, Gly, Ala, His, Arg, Cys, and Tyr) in the Tibetan chicken from different cities are shown in Table S2. These components include total amino acids (TAA), essential amino acids (EAA) and flavor amino acids (FAA). The concentrations of the amino acids in Tibetan chicken were similar to those reported previously [23]. The mean content of the TAA of Tibetan chicken in all cities was 22.06 g/100 g. The mean content of Glu (3.45 g/100 g) and Asp (2.19 g/100 g) was found to be the highest in the four cities’ Tibetan chicken samples, followed by Lys (2.14 g/100 g) and Leu (1.98 g/100 g), whereas Cys (0.23 g/100 g), Met (0.57 g/100 g), and Tyr (0.76 g/100 g) were the lowest.

The mean content of the FAA in the Tibetan chicken samples was 11.43 g/100 g. There are three types of FAA in Tibetan chicken, namely umami amino acids (Asp, Pro, and Glu), sweet amino acids (Gly, Ala, and Ser), and aromatic amino acids (Phe and Tyr). Among them, the average content range of umami amino acids Glu (3.45 g/100 g) and Asp (2.19 g/100 g) are the highest. The content of FAA determines the flavor of the chicken; the higher the content of FAA, the more intense the flavor of chicken [24]. In this study, the FAA of Tibetan chicken account for 51.75–51.88% of the total amino acids (Table S3), which is much higher than that of chicken breeds in other regions [25].

As for the nutritional indicators of Tibetan chicken, the Tibetan chicken sample contains 7 kinds of human essential amino acids (Phe, Met, Lys, Thr, Leu, Ile, Val). The mean concentration was 8.94 g/100 g, thus accounting for 40.31–40.83% of the TAA (Table S3), which is higher than the recommended values of essential amino acids for humans, as per the World Health Organization and the Food and Agriculture Organization of the United Nations (WHO/FAO) (40.00%). Moreover, the recommended values of essential amino acids for humans according to the WHO/FAO are as follows: 5.00% Val/TAA, 4.00% Ile/TAA, 7.00% Leu/TAA, 4.00% Thr/TAA, and 40% EAA/TAA [26]. As shown in Table S3, the values of all of the essential amino acids and the EAA/TAA in the Tibetan chicken reached the ideal amino acid standard as stipulated by the WHO/FAO, thereby suggesting that Tibetan chicken contains an abundant and appropriate proportion. Moreover, Zhang et al. measured the content and composition of amino acids in 30 cocks and 30 hens from three strains of Aba Tibetan chicken in order to study the nutritional value of the muscles of Aba Tibetan chicken [23]. The values of the essential amino acids and the EAA/TAA in the Aba Tibetan chicken were as follows: 4.45% Val/TAA, 3.94% Ile/TAA, 8.28% Leu/TAA, 4.80% Thr/TAA, and 37.64% EAA/TAA. Thus, comparing the values of the essential amino acids and the EAA/TAA between Tibetan chicken from Tibet and Aba, we can conclude that the Tibetan chicken from Tibet has a higher nutritional value.

### 3.2. Differences in Amino Acids in Different Regions

As shown in Table S2, all of the amino acid concentrations of Tibetan chicken in the four regions were significantly different (*p* < 0.05), according to the post-hoc Duncan’s multiple comparisons of ANOVA testing. The EAA and FAA percentages in the Tibetan chicken were in the order of Lhase > Linzhi > Xigaze > Shannan, and the contents of the EAA in the Tibetan chicken samples were in the order of Linzhi > Lhase > Xigaze > Shannan. The contents of Phe, Lys, Leu, Ile, Val, Glu, Pro, Arg, and Tyr in the Tibetan chicken samples in the Linzhi area are higher than those in the other three cities. In addition, the contents of Met, Thr, Asp, Ser, Gly, Ala, and His in the Tibetan chicken in Lhasa are the highest among the four areas. Conversely, the percentage composition of Phe, Lys, Thr, Leu, Ile, Val, Asp, Ser, Glu, Pro, Gly, Ala, His, Arg, and Tyr of the Tibetan chicken samples in Shannan was the lowest among the four regions. What deserves special attention, however, is Cys, which is different from other amino acids and has the lowest content (0.22 g/100 g) in the Lhasa Tibetan chickens and the highest content (0.25 g/100 g) in the Xigaze Tibetan chickens. In summary, except for the Cys of the non-essential amino acids, all of the amino acid concentrations in the Tibetan chicken samples from Lhasa and Linzhi were significantly higher than those from Shannan and Xigaze.

The increase in altitude leads to changes in the various environmental parameters of the Qinghai–Tibet Plateau, such as the decrease in oxygen content, temperature, and rainfall [6]. Changes in environmental parameters lead to changes in the composition of amino acids in animal-derived foods [27]. In general, the amino acid content in Tibetan chicken increased at first, then decreased with the increase in altitude. Coincidentally, Zhu et al. analyzed the amino acid content of yak meat samples collected from the Qinghai–Tibet Plateau at altitudes of 3169 m and 4020 m and found that some amino acid contents first increased and then decreased with the elevation [28]. It can be inferred that the amino acid content in the muscle of high-altitude animals above 3500 m, undergoes a special change with respect to altitude.

### 3.3. Chemometric Analysis of Tibetan Chickens in Different Regions of Tibet

**Figure 2:**
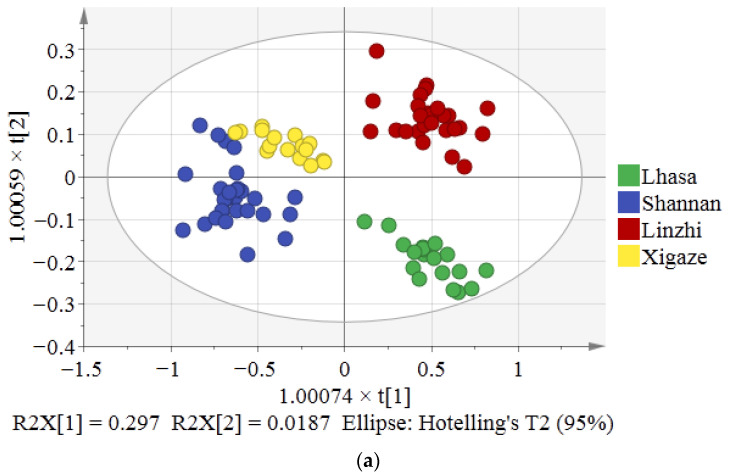

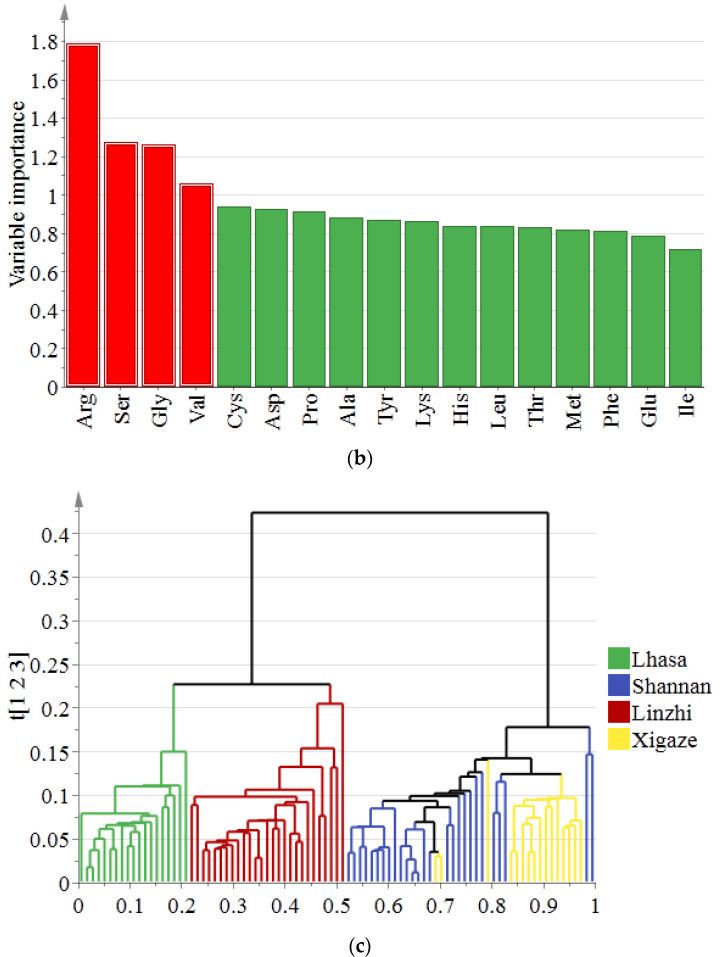
The OPLS-DA plot of Tibetan chicken from four regions with the data on amino acids (**a**); the variable importance projection of Tibetan chicken samples from four regions of Tibet with data on amino acids (**b**); the HCA plot of amino acids in Tibetan chicken from four cities (**c**).

To make a preliminary assessment, based on amino acids, of the reliability of a multivariate separation for classifying the four regions where Tibetan chicken in Tibet are present, discriminant analyses were performed. All of the seventeen amino acids in the Tibetan chicken samples were analyzed by OPLS-DA (Figure 2a). The OPLS-DA model was validated by 7-fold cross-validation, which is an internal prediction validation method wherein the parameters R2 (cumulative) and Q2 (cumulative) define the accumulated explained variance and derived model predictability, respectively. These were then used to assess the OPLS-DA model’s performance [29]. In Figure 2a, the values of R2Y and Q2 of the OPLS-DA model are 0.733 and 0.587, respectively, thus satisfying R2Y > Q2 > 0.50— which indicates that the model is adequate for fitting and prediction [30]. The OPLS-DA score plots of the four cities could bind the data into four classes, based on amino acids: Linzhi samples (red dots) and Lhasa samples (green dots) could almost be separated from the other samples, but the chickens from Shannan (blue dots) and Xigaze (yellow dots) overlapped. The variable influence on projection (VIP) value (Figure 2b), as calculated by the OPLS-DA model, could quantify the contribution of each variable to the classification. The greater the VIP value, the more significant the difference of variables among the different regions where Tibetan chicken are present [31]. There were four variables (Arg, Ser, Gly, and Val) that had higher importance values (>1). These variables were used in order to distinguish the Tibetan chicken in the four areas. Tang et al. found that Arg, Gly, and Ser are the main amino acids that can be used to distinguish musk at different altitudes [32]. Moreover, Arg and Val are differential markers that can be utilized to distinguish the Jersey cattle that are raised at different altitudes [33]. Thus, these four amino acids of Arg, Ser, Gly, and Val were associated with important altitude metabolic pathways. In addition, the results obtained after HCA are displayed as a dendrogram (Figure 2c), and all samples were grouped based on similarity. There are four well-defined clusters are visible, and just three Xigaze samples (yellow lines) were wrongly classified into Shannan samples (blue lines). The results of the HCA were similar to the OPLS-DA score plots, and the two discrimination results verify each other.

Next, the LDA model, construed via a by “leave-one-out” cross-validation discriminant analysis, was used to determine whether the Tibetan chicken samples from various sources could be classified by all amino acids. As shown in Table 2, the cross-discrimination rate of amino acid indexes in Tibetan chicken was 94.4%, and the original discrimination rate was 93.3%. In the original classification, there were five Shannan samples that were misclassified into Xigaze samples, and the accuracy rate regarding this was 81.5%. Similarly, six Shannan samples were misjudged to be Xigaze samples in the cross-validation, where the accuracy rate was 77.8%. This was consistent with our previous results. Compared with our previous research [8], when using a stable isotope technique to distinguish Tibetan chicken samples from the four cities in Tibet, the highest cross-validation rate is only 61.0%, which is far lower than those using amino acids. It is indicated that amino acid was the most effective method to improve the accuracy, and also provided a good idea for tracking the Tibetan chickens in Tibet.

### 3.4. Correlation between Amino Acids and Altitude

The relationship between the amino acid contents (Phe, Met, Lys, Thr, Leu, Ile, Val, Asp, Ser, Glu, Pro, Gly, Ala, His, Arg, Cys, Tyr, EAA, FAA, and TAA) of the Tibetan chicken, with the different altitudes of the four cities (Lhasa, Shannan, Linzhi, and Xigaze) in Tibet are shown in Figure S1. All of the amino acids of Tibetan chicken are influenced by altitudes and are normally distributed in line with the altitudes. Except for the opposite trend of Cys, all amino acids in the Tibetan chicken, including EAA, FAA, and TAA, showed a single peak trend of first increasing and then decreasing with the elevation. At an altitude of 3850 m, most of the amino acid contents in Tibetan chickens reached the inflection point of the curve. Moreover, when conducting the multivariate statistical analysis, it was found that some Shannan samples were misjudged as Xigaze. This was because the samples obtained from the altitudes of Xigaze (3995 m) and Shannan (3660 m) are in a symmetrical position with respect to the axis (3850 m), which leads to similar amino acid content in the Tibetan chicken of the two cities. That is to say, this method cannot distinguish the Tibetan chicken samples in the cities with an axisymmetric altitude.

The reasons for the above phenomenon are mainly explainable in two aspects. On one hand, the Tibetan chicken grows well at high altitude areas because it has strong physiological adaptability to both the cold and low oxygen plateau environments [4]. In the long-term adaptation to a low-oxygen plateau environment, the blood circulation system and body tissues of plateau animals use many compensatory mechanisms in order to combine with oxygen efficiently, which is demonstrated by increasing hemoglobin and myoglobin. When this occurs, the blood viscosity reduces, blood flow velocity accelerates, and the hemodynamic change caused by increasing erythrocyte is relieved, thus resulting in the oxygen-carrying capacity being improved [34,35]. Hemoglobin and myoglobin are composed of many kinds of amino acids. To some extent, the increase in hemoglobin and myoglobin in the Tibetan chicken with respect to altitude means that the amino acid content in Tibetan chicken also increases with altitude. Moreover, there were four variables (Arg, Ser, Gly, and Val) with a VIP > 1 that were considered significant with respect to distinguishing the Tibetan chicken from the four areas. Among them, Arg is involved in the NO pathway, which is the only substrate for NO production. It can be used as a drug to treat high-altitude pulmonary edema [36]. Reduced glutathione (GSH), which is an important antioxidant synthesized from Glu, Cyst, and Gly, is necessary to prevent hypoxia-induced inflammation [37]. Thus, the organisms of Tibetan chicken can better adapt to the high-altitude ecological environment by regulating the contents of the above amino acids. However, there are no references that can explain the reason for stress physiological adaptability and why the amino acid content in plateau animals decreases when it exceeds a certain altitude of meters. It is suspected that after reaching a certain altitude, the harsh environment of cold and low oxygen on the plateau intensifies, which renders the Tibetan chicken unable to be adapt, thus resulting in the decrease in amino acid content.

On the other hand, it was found that a low-protein diet resulted in low protein in Tibetan chicken. This is because the less protein chickens take in, the less energy is decomposed and recombined protein consumed, and the more fat is deposited in the form of fat. In addition, when the content of some amino acids in the diet is low, chickens need to increase their feed intake to meet their requirements for restricted amino acids, which will lead to excessive energy intake, thereby resulting in an increase in body fat content and a corresponding decrease in protein content [38,39]. The Tibetan chicken is free-range and raised naturally without any supplementary feeding, such that they are good at foraging and eating all kinds of local forage that they can find [4]. Therefore, the amino acid content of Tibetan chickens is positively correlated with the protein content in local forage. Kering et al. found that the nitrogen application (urea and ammonium nitrate) increased the crude protein of both spring- and summer-harvested forage [40]. That is when the nitrogen application rate was 224 kg/ha, which is where the crude protein of spring forage and summer forage increased by 78% and 56%, respectively. This means that the protein content of forage grass in the Qinghai–Tibet Plateau is related to the nitrogen content in the soil. Moreover, plants can use not only inorganic nitrogen (NH^4+^ and NO^3−^), but also organic nitrogen (amino acids and urea, etc.) [41]. When organic nitrogen concentration is equal to or higher than inorganic nitrogen, plants can directly use organic nitrogen, such as free amino acids in the soil as a nitrogen source [42]. The special climate of high altitude and low temperature on the Qinghai–Tibet Plateau restricts soil mineralization, thus resulting in the soil organic nitrogen content exceeding the mineral nitrogen content [43]. Therefore, the nitrogen of high-altitude herbage on the Qinghai–Tibet Plateau comes from organic nitrogen, as well as inorganic nitrogen. That is to say, the higher the content of total nitrogen (inorganic nitrogen and organic nitrogen) in soil, the higher the protein content of herbage on the Qinghai–Tibet Plateau. According to study [44], at the altitude of 3105 m to 4306 m, the soil total nitrogen content in the Qinghai–Tibet Plateau first increased and then decreased with the altitude, reaching the peak at about 3850 m. This phenomenon is synchronized with the change in of amino acid content in Tibetan chicken with altitude. In brief, the change in altitude leads to the change in total nitrogen content in the soil, which leads to the change in protein content in the pasture, and then to the corresponding change in amino acid content in Tibetan chickens.

## 4. Conclusions

In this paper, amino acid profiling (Phe, Met, Lys, Thr, Leu, Ile, Val, Asp, Ser, Glu, Pro, Gly, Ala, His, Arg, Cys, and Tyr) was used to identify the Tibetan chicken samples from four cities in Tibet. The results showed that all of the amino acid concentrations of Tibetan chicken in the four regions were significantly different. Furthermore, the cross-discrimination rate was 94.4%, and the original discrimination rate was 93.3%. Moreover, we found that the amino acid concentrations of Tibetan chicken are closely related to altitudes. Except for Cys, all amino acids in Tibetan chickens showed a normal distribution trend of first increasing and then decreasing with the increase in altitude. There were four variables (Arg, Ser, Gly, and Val) with a VIP of >1 that were considered significant in distinguishing Tibetan chicken from the four areas. In summary, this study first applied amino acid profiling combined with chemometrics to the traceability of Tibetan chicken, which will provide a new idea for the traceability of plateau animal foods.

## Figures and Tables

**Table 1:** Region information of Tibetan chicken samples.

City	Number	Longitude/°E	Latitude/°N	Average Altitude/m	Mean Annual Temperature/°C
Lhasa	19	90.11 to 90.69	29.28 to 29.50	3817	7.4
Shannan	27	91.40 to 92.49	29.15 to 29.30	3660	8.8
Linzhi	27	91.03 to 92.90	29.65 to 30.02	3700	8.7
Xigaze	16	88.27 to 88.43	29.06 to 29.44	3995	6.3

**Table 2:** Classification of Tibetan chicken samples based on amino acids by LDA.

			Predicted Group Membership ^a^				Total
		Region	Lhasa	Shannan	Linzhi	Xigaze	
Original	Count	Lhasa	19	0	0	0	19
		Shannan	0	22	0	5	27
		Linzhi	0	0	27	0	27
		Xigaze	0	0	0	16	16
	%		100.0	81.5	100.0	100.0	94.4 ^b^
Cross-validation	Count	Lhasa	19	0	0	0	19
		Shannan	0	21	0	6	27
		Linzhi	0	0	27	0	27
		Xigaze	0	0	0	16	16
	%		100.0	77.8	100.0	100.0	93.3 ^c^

^a^ The number of correctly classified observations are tabulated diagonally. ^b^ 94.4% of empirical grouped observations correctly classified. ^c^ 93.3% of cross-validated grouped observations correctly classified.

## Data Availability

The data are available from the corresponding author.

## Supplementary Materials

The following supporting information can be downloaded at: https://www.mdpi.com/article/10.3390/foods12040876/s1, Figure S1: Relationship between means amino acids concentration and altitude of different cities; Table S1: The data employed for multivariate analysis; Table S2: Composition and concentrations of seventeen amino acids in Tibetan chicken from different cities; Table S3: Comparision of amino acids with the amino acid pattern profile recommended by the World Health Organization and the Food and Agriculture Organization of the United Nations (WHO/FAO).
