# Supplementary material for: A New and Effective Method to Trace Tibetan Chicken by Amino Acid Profiling
Source: Foods. 2023 Feb 18;12(4):876. doi: 10.3390/foods12040876 (PMC9957330; doi:10.3390/foods12040876)
Supplement: Supplementary file 1 [file foods-12-00876-s001.zip › foods-2172667-supplementary.pdf]

**Table S1.** The data employed for multivariate analysis.

| Number | Region | Phe g/100 g | Met g/100 g | Lys g/100 g | Thr g/100 g | Leu g/100 g | Ile g/100 g | Val g/100 g | Asp g/100 g | Ser g/100 g | Glu g/100 g | Pro g/100 g | Gly g/100 g | Ala g/100g | His g/100 g | Arg g/100 g | Cys g/100 g | Tyr g/100 g |
|---|---|---|---|---|---|---|---|---|---|---|---|---|---|---|---|---|---|---|
| 1 | Lhasa | 1.00 | 0.73 | 2.33 | 1.18 | 2.12 | 1.23 | 1.29 | 2.46 | 0.99 | 3.81 | 0.87 | 1.12 | 1.49 | 1.03 | 1.68 | 0.24 | 0.84 |
| 2 | Lhasa | 0.98 | 0.71 | 2.28 | 1.16 | 2.08 | 1.18 | 1.24 | 2.40 | 0.98 | 3.69 | 0.86 | 1.06 | 1.44 | 1.01 | 1.62 | 0.23 | 0.84 |
| 3 | Lhasa | 0.87 | 0.60 | 2.05 | 1.04 | 1.84 | 1.03 | 1.11 | 2.12 | 0.88 | 3.32 | 0.78 | 0.97 | 1.30 | 0.90 | 1.45 | 0.23 | 0.69 |
| 4 | Lhasa | 0.89 | 0.44 | 2.08 | 1.05 | 1.89 | 1.06 | 1.12 | 2.16 | 0.87 | 3.40 | 0.76 | 1.00 | 1.35 | 0.85 | 1.46 | 0.23 | 0.72 |
| 5 | Lhasa | 0.99 | 0.47 | 2.28 | 1.19 | 2.09 | 1.18 | 1.32 | 2.43 | 1.00 | 3.62 | 0.92 | 1.18 | 1.55 | 1.05 | 1.60 | 0.25 | 0.78 |
| 6 | Lhasa | 0.88 | 0.59 | 2.01 | 1.04 | 1.84 | 1.03 | 1.12 | 2.15 | 0.89 | 3.35 | 0.93 | 1.28 | 1.43 | 0.84 | 1.52 | 0.23 | 0.71 |
| 7 | Lhasa | 1.06 | 0.74 | 2.45 | 1.27 | 2.21 | 1.24 | 1.36 | 2.57 | 1.12 | 3.97 | 1.01 | 1.32 | 1.63 | 1.09 | 1.81 | 0.29 | 0.86 |
| 8 | Lhasa | 1.08 | 0.76 | 2.48 | 1.29 | 2.25 | 1.27 | 1.37 | 2.64 | 1.12 | 4.00 | 0.96 | 1.17 | 1.59 | 1.11 | 1.78 | 0.27 | 0.89 |
| 9 | Lhasa | 0.97 | 0.66 | 2.25 | 1.15 | 2.02 | 1.12 | 1.21 | 2.32 | 0.98 | 3.61 | 0.93 | 1.18 | 1.48 | 1.01 | 1.63 | 0.26 | 0.77 |
| 10 | Lhasa | 1.01 | 0.71 | 2.34 | 1.19 | 2.11 | 1.19 | 1.25 | 2.45 | 0.99 | 3.79 | 0.90 | 1.11 | 1.48 | 1.06 | 1.65 | 0.22 | 0.84 |
| 11 | Lhasa | 0.96 | 0.66 | 2.21 | 1.11 | 1.98 | 1.14 | 1.20 | 2.33 | 0.93 | 3.60 | 0.83 | 1.03 | 1.39 | 0.97 | 1.57 | 0.17 | 0.78 |
| 12 | Lhasa | 1.17 | 0.81 | 2.74 | 1.37 | 2.48 | 1.45 | 1.49 | 2.83 | 1.14 | 4.32 | 0.99 | 1.24 | 1.69 | 1.22 | 1.95 | 0.21 | 1.02 |
| 13 | Lhasa | 0.90 | 0.66 | 2.12 | 1.07 | 1.93 | 1.12 | 1.15 | 2.21 | 0.90 | 3.43 | 0.77 | 0.98 | 1.33 | 0.91 | 1.50 | 0.20 | 0.77 |
| 14 | Lhasa | 0.94 | 0.68 | 2.24 | 1.13 | 2.02 | 1.18 | 1.22 | 2.34 | 0.95 | 3.62 | 0.82 | 1.04 | 1.40 | 0.97 | 1.56 | 0.16 | 0.82 |
| 15 | Lhasa | 1.00 | 0.73 | 2.34 | 1.16 | 2.12 | 1.23 | 1.25 | 2.41 | 0.97 | 3.79 | 0.86 | 1.06 | 1.45 | 1.04 | 1.62 | 0.20 | 0.85 |
| 16 | Lhasa | 0.95 | 0.70 | 2.25 | 1.13 | 2.04 | 1.17 | 1.18 | 2.33 | 0.94 | 3.69 | 0.82 | 1.02 | 1.39 | 0.97 | 1.57 | 0.19 | 0.83 |
| 17 | Lhasa | 0.88 | 0.62 | 2.06 | 1.04 | 1.86 | 1.08 | 1.12 | 2.15 | 0.87 | 3.26 | 0.77 | 0.96 | 1.29 | 0.87 | 1.45 | 0.18 | 0.76 |
| 18 | Lhasa | 0.83 | 0.57 | 1.96 | 0.98 | 1.76 | 1.02 | 1.03 | 2.02 | 0.82 | 3.25 | 0.75 | 0.97 | 1.22 | 0.80 | 1.40 | 0.19 | 0.72 |
| 19 | Lhasa | 0.89 | 0.64 | 2.10 | 1.05 | 1.90 | 1.11 | 1.12 | 2.17 | 0.87 | 3.39 | 0.78 | 0.96 | 1.30 | 0.71 | 1.47 | 0.16 | 0.76 |
| 20 | Shannan | 0.83 | 0.61 | 1.98 | 1.01 | 1.84 | 1.02 | 1.05 | 2.06 | 0.82 | 3.17 | 0.76 | 0.94 | 1.31 | 0.83 | 1.39 | 0.27 | 0.67 |
| 21 | Shannan | 0.51 | 0.27 | 1.18 | 0.60 | 1.08 | 0.60 | 0.62 | 1.16 | 0.52 | 1.88 | 0.51 | 0.69 | 0.81 | 0.50 | 0.86 | 0.15 | 0.35 |
| 22 | Shannan | 0.80 | 0.34 | 1.88 | 0.94 | 1.74 | 0.97 | 0.98 | 1.89 | 0.78 | 2.98 | 0.71 | 0.88 | 1.23 | 0.79 | 1.30 | 0.22 | 0.58 |

| | | | | | | | | | | | | | | | | | | |
|---|---|---|---|---|---|---|---|---|---|---|---|---|---|---|---|---|---|---|
| 23 | Shannan | 0.81 | 0.55 | 1.90 | 0.96 | 1.76 | 0.98 | 1.02 | 1.90 | 0.82 | 3.02 | 0.77 | 0.99 | 1.29 | 0.84 | 1.36 | 0.25 | 0.59 |
| 24 | Shannan | 0.85 | 0.39 | 1.99 | 1.00 | 1.86 | 1.05 | 1.03 | 2.03 | 0.84 | 3.24 | 0.77 | 0.97 | 1.31 | 0.73 | 1.40 | 0.23 | 0.65 |
| 25 | Shannan | 0.89 | 0.64 | 2.08 | 1.06 | 1.92 | 1.08 | 1.11 | 2.19 | 0.88 | 3.37 | 0.77 | 0.97 | 1.36 | 0.96 | 1.45 | 0.26 | 0.67 |
| 26 | Shannan | 0.86 | 0.53 | 1.98 | 1.00 | 1.83 | 1.04 | 1.05 | 2.08 | 0.83 | 3.19 | 0.75 | 0.95 | 1.31 | 0.75 | 1.37 | 0.26 | 0.63 |
| 27 | Shannan | 0.88 | 0.66 | 2.06 | 1.04 | 1.93 | 1.09 | 1.09 | 2.15 | 0.85 | 3.28 | 0.78 | 0.96 | 1.36 | 0.85 | 1.45 | 0.24 | 0.74 |
| 28 | Shannan | 0.90 | 0.51 | 2.12 | 1.06 | 1.94 | 1.10 | 1.10 | 2.20 | 0.88 | 3.39 | 0.78 | 0.97 | 1.38 | 0.87 | 1.45 | 0.26 | 0.67 |
| 29 | Shannan | 0.86 | 0.59 | 2.00 | 1.01 | 1.85 | 1.06 | 1.06 | 2.07 | 0.86 | 3.09 | 0.77 | 0.94 | 1.28 | 0.81 | 1.40 | 0.23 | 0.68 |
| 30 | Shannan | 1.05 | 0.70 | 2.43 | 1.22 | 2.24 | 1.26 | 1.30 | 2.53 | 1.05 | 3.86 | 0.93 | 1.15 | 1.57 | 0.95 | 1.69 | 0.32 | 0.85 |
| 31 | Shannan | 0.79 | 0.29 | 1.84 | 0.93 | 1.71 | 0.97 | 0.95 | 1.91 | 0.77 | 2.92 | 0.71 | 0.87 | 1.19 | 0.71 | 1.27 | 0.22 | 0.58 |
| 32 | Shannan | 0.79 | 0.36 | 1.86 | 0.92 | 1.71 | 0.97 | 0.95 | 1.91 | 0.76 | 2.96 | 0.70 | 0.86 | 1.19 | 0.67 | 1.31 | 0.21 | 0.59 |
| 33 | Shannan | 0.88 | 0.63 | 2.01 | 1.02 | 1.89 | 1.07 | 1.07 | 2.12 | 0.84 | 3.25 | 0.80 | 1.01 | 1.34 | 0.82 | 1.44 | 0.23 | 0.70 |
| 34 | Shannan | 0.96 | 0.67 | 2.20 | 1.11 | 2.05 | 1.16 | 1.14 | 2.31 | 0.93 | 3.54 | 0.91 | 1.18 | 1.45 | 0.86 | 1.60 | 0.22 | 0.77 |
| 35 | Shannan | 0.90 | 0.48 | 2.07 | 1.05 | 1.94 | 1.10 | 1.10 | 2.20 | 0.88 | 3.36 | 0.84 | 1.05 | 1.39 | 0.84 | 1.49 | 0.25 | 0.70 |
| 36 | Shannan | 0.93 | 0.44 | 2.09 | 1.07 | 1.96 | 1.12 | 1.13 | 2.22 | 0.86 | 3.33 | 0.82 | 1.01 | 1.39 | 0.82 | 1.46 | 0.26 | 0.67 |
| 37 | Shannan | 0.89 | 0.63 | 2.04 | 1.04 | 1.90 | 1.09 | 1.12 | 2.16 | 0.87 | 3.28 | 0.80 | 0.99 | 1.37 | 0.86 | 1.46 | 0.22 | 0.68 |
| 38 | Shannan | 0.88 | 0.63 | 2.00 | 1.00 | 1.87 | 1.06 | 1.05 | 2.09 | 0.82 | 3.27 | 0.81 | 1.02 | 1.33 | 0.75 | 1.47 | 0.22 | 0.70 |
| 39 | Shannan | 0.89 | 0.43 | 2.03 | 0.99 | 1.84 | 1.05 | 1.02 | 2.07 | 0.82 | 3.29 | 0.79 | 0.97 | 1.30 | 0.73 | 1.42 | 0.23 | 0.65 |
| 40 | Shannan | 0.83 | 0.38 | 1.90 | 0.96 | 1.77 | 1.01 | 0.99 | 2.02 | 0.80 | 3.14 | 0.72 | 0.87 | 1.23 | 0.70 | 1.34 | 0.23 | 0.61 |
| 41 | Shannan | 0.84 | 0.59 | 1.93 | 0.97 | 1.79 | 1.04 | 1.06 | 2.01 | 0.79 | 3.04 | 0.74 | 0.90 | 1.27 | 0.72 | 1.36 | 0.26 | 0.65 |
| 42 | Shannan | 0.83 | 0.60 | 1.99 | 0.99 | 1.90 | 1.06 | 1.08 | 2.05 | 0.83 | 3.25 | 0.79 | 1.00 | 1.33 | 0.74 | 1.44 | 0.29 | 0.69 |
| 43 | Shannan | 0.83 | 0.33 | 1.97 | 0.97 | 1.87 | 1.05 | 1.04 | 1.99 | 0.81 | 3.17 | 0.77 | 0.96 | 1.29 | 0.70 | 1.38 | 0.25 | 0.64 |
| 44 | Shannan | 0.95 | 0.68 | 2.19 | 1.11 | 2.08 | 1.16 | 1.15 | 2.30 | 0.93 | 3.66 | 0.87 | 1.11 | 1.45 | 0.82 | 1.60 | 0.25 | 0.79 |
| 45 | Shannan | 0.81 | 0.50 | 1.87 | 0.93 | 1.78 | 0.98 | 0.98 | 1.91 | 0.79 | 3.05 | 0.80 | 1.01 | 1.25 | 0.68 | 1.37 | 0.26 | 0.63 |
| 46 | Shannan | 0.86 | 0.55 | 2.01 | 1.00 | 1.93 | 1.07 | 1.07 | 2.06 | 0.83 | 3.26 | 0.81 | 1.03 | 1.34 | 0.82 | 1.45 | 0.29 | 0.70 |
| 47 | Linzhi | 0.90 | 0.66 | 2.22 | 1.10 | 2.04 | 1.17 | 1.25 | 2.29 | 0.94 | 3.60 | 0.84 | 1.02 | 1.38 | 0.91 | 1.60 | 0.19 | 0.82 |
| 48 | Linzhi | 0.97 | 0.63 | 2.27 | 1.11 | 2.09 | 1.18 | 1.24 | 2.28 | 0.94 | 3.66 | 0.88 | 1.05 | 1.40 | 0.83 | 1.62 | 0.23 | 0.86 |

| | | | | | | | | | | | | | | | | | | |
|---|---|---|---|---|---|---|---|---|---|---|---|---|---|---|---|---|---|---|
| 49 | Linzhi | 1.01 | 0.71 | 2.57 | 1.25 | 2.33 | 1.33 | 1.40 | 2.56 | 1.05 | 4.09 | 0.94 | 1.12 | 1.54 | 1.02 | 1.82 | 0.22 | 0.97 |
| 50 | Linzhi | 0.91 | 0.69 | 2.27 | 1.12 | 2.11 | 1.19 | 1.25 | 2.28 | 0.94 | 3.68 | 0.82 | 0.98 | 1.37 | 0.87 | 1.66 | 0.23 | 0.85 |
| 51 | Linzhi | 0.92 | 0.66 | 2.22 | 1.10 | 2.04 | 1.16 | 1.21 | 2.22 | 0.93 | 3.62 | 0.85 | 1.02 | 1.37 | 0.80 | 1.61 | 0.23 | 0.81 |
| 52 | Linzhi | 1.04 | 0.75 | 2.56 | 1.27 | 2.37 | 1.36 | 1.41 | 2.55 | 1.08 | 4.11 | 0.99 | 1.19 | 1.57 | 0.95 | 1.85 | 0.25 | 0.96 |
| 53 | Linzhi | 0.91 | 0.66 | 2.27 | 1.10 | 2.08 | 1.18 | 1.21 | 2.24 | 0.93 | 3.64 | 0.83 | 0.98 | 1.37 | 0.77 | 1.60 | 0.20 | 0.82 |
| 54 | Linzhi | 1.03 | 0.45 | 2.53 | 1.23 | 2.33 | 1.32 | 1.39 | 2.51 | 1.04 | 3.99 | 0.93 | 1.13 | 1.54 | 1.01 | 1.73 | 0.23 | 0.89 |
| 55 | Linzhi | 0.96 | 0.65 | 2.41 | 1.16 | 2.18 | 1.25 | 1.32 | 2.34 | 0.98 | 3.78 | 0.88 | 1.05 | 1.46 | 1.00 | 1.68 | 0.25 | 0.89 |
| 56 | Linzhi | 1.03 | 0.57 | 2.06 | 1.00 | 1.87 | 1.07 | 1.09 | 2.00 | 0.87 | 3.40 | 0.82 | 0.94 | 1.25 | 0.66 | 1.49 | 0.20 | 0.78 |
| 57 | Linzhi | 0.96 | 0.55 | 1.82 | 0.90 | 1.68 | 0.96 | 1.01 | 1.87 | 0.80 | 3.01 | 0.95 | 1.23 | 1.26 | 0.62 | 1.44 | 0.20 | 0.70 |
| 58 | Linzhi | 1.05 | 0.58 | 2.06 | 1.00 | 1.84 | 1.05 | 1.09 | 2.05 | 0.89 | 3.34 | 0.85 | 0.98 | 1.23 | 0.69 | 1.47 | 0.24 | 0.77 |
| 59 | Linzhi | 1.35 | 0.64 | 2.61 | 1.30 | 2.44 | 1.36 | 1.41 | 2.66 | 1.12 | 4.37 | 1.05 | 1.27 | 1.62 | 0.82 | 1.79 | 0.26 | 0.90 |
| 60 | Linzhi | 1.06 | 0.72 | 2.47 | 1.22 | 2.30 | 1.30 | 1.37 | 2.49 | 1.02 | 4.02 | 0.97 | 1.12 | 1.54 | 0.75 | 1.78 | 0.27 | 0.96 |
| 61 | Linzhi | 0.97 | 0.41 | 2.32 | 1.13 | 2.12 | 1.19 | 1.26 | 2.32 | 0.95 | 3.78 | 0.84 | 0.97 | 1.39 | 0.80 | 1.62 | 0.21 | 0.83 |
| 62 | Linzhi | 1.03 | 0.73 | 2.53 | 1.22 | 2.31 | 1.31 | 1.37 | 2.52 | 1.01 | 4.04 | 0.95 | 1.09 | 1.54 | 0.89 | 1.81 | 0.25 | 0.99 |
| 63 | Linzhi | 0.94 | 0.67 | 2.30 | 1.11 | 2.08 | 1.19 | 1.27 | 2.29 | 0.94 | 3.65 | 0.88 | 1.06 | 1.43 | 0.81 | 1.62 | 0.21 | 0.88 |
| 64 | Linzhi | 1.00 | 0.69 | 2.39 | 1.16 | 2.20 | 1.23 | 1.28 | 2.40 | 0.98 | 3.78 | 0.91 | 1.08 | 1.45 | 0.84 | 1.70 | 0.25 | 0.94 |
| 65 | Linzhi | 0.88 | 0.67 | 2.13 | 1.06 | 1.99 | 1.12 | 1.19 | 2.17 | 0.89 | 3.47 | 0.80 | 0.96 | 1.34 | 0.89 | 1.53 | 0.21 | 0.83 |
| 66 | Linzhi | 0.93 | 0.45 | 2.23 | 1.08 | 2.08 | 1.16 | 1.20 | 2.25 | 0.92 | 3.66 | 0.89 | 1.09 | 1.40 | 0.85 | 1.59 | 0.23 | 0.82 |
| 67 | Linzhi | 0.85 | 0.64 | 2.06 | 1.02 | 1.91 | 1.08 | 1.13 | 2.09 | 0.85 | 3.32 | 0.80 | 0.95 | 1.29 | 0.86 | 1.48 | 0.23 | 0.81 |
| 68 | Linzhi | 0.91 | 0.68 | 2.19 | 1.10 | 2.01 | 1.13 | 1.23 | 2.24 | 0.92 | 3.59 | 0.83 | 1.04 | 1.40 | 0.86 | 1.57 | 0.25 | 0.84 |
| 69 | Linzhi | 0.95 | 0.70 | 2.30 | 1.12 | 2.11 | 1.17 | 1.21 | 2.29 | 0.93 | 3.69 | 0.89 | 1.05 | 1.39 | 0.86 | 1.63 | 0.26 | 0.89 |
| 70 | Linzhi | 0.93 | 0.68 | 2.24 | 1.10 | 2.08 | 1.15 | 1.19 | 2.26 | 0.92 | 3.62 | 0.87 | 1.06 | 1.39 | 0.84 | 1.57 | 0.23 | 0.88 |
| 71 | Linzhi | 0.84 | 0.59 | 2.01 | 0.99 | 1.83 | 1.03 | 1.09 | 2.03 | 0.85 | 3.17 | 0.80 | 0.95 | 1.24 | 0.73 | 1.42 | 0.19 | 0.80 |
| 72 | Linzhi | 0.89 | 0.63 | 2.13 | 1.05 | 1.96 | 1.11 | 1.18 | 2.17 | 0.90 | 3.38 | 0.83 | 1.01 | 1.34 | 0.75 | 1.55 | 0.21 | 0.86 |
| 73 | Linzhi | 0.92 | 0.64 | 2.21 | 1.08 | 2.01 | 1.16 | 1.24 | 2.22 | 0.92 | 3.44 | 0.86 | 1.03 | 1.38 | 0.79 | 1.56 | 0.22 | 0.87 |
| 74 | Xigaze | 0.90 | 0.36 | 2.14 | 1.06 | 2.03 | 1.13 | 1.13 | 2.17 | 0.88 | 3.51 | 0.82 | 1.04 | 1.40 | 0.84 | 1.53 | 0.27 | 0.72 |

| | | | | | | | | | | | | | | | | | | |
|---|---|---|---|---|---|---|---|---|---|---|---|---|---|---|---|---|---|---|
| 75 | Xigaze | 0.87 | 0.58 | 2.09 | 1.03 | 1.99 | 1.11 | 1.13 | 2.13 | 0.87 | 3.35 | 0.82 | 1.04 | 1.39 | 0.84 | 1.51 | 0.29 | 0.73 |
| 76 | Xigaze | 0.88 | 0.38 | 2.09 | 1.02 | 1.95 | 1.09 | 1.10 | 2.07 | 0.87 | 3.32 | 0.79 | 0.99 | 1.36 | 0.73 | 1.47 | 0.28 | 0.65 |
| 77 | Xigaze | 0.88 | 0.41 | 2.09 | 1.01 | 1.99 | 1.11 | 1.10 | 2.09 | 0.85 | 3.30 | 0.80 | 1.01 | 1.37 | 0.82 | 1.48 | 0.25 | 0.71 |
| 78 | Xigaze | 0.87 | 0.36 | 2.12 | 1.03 | 2.01 | 1.13 | 1.11 | 2.16 | 0.86 | 3.33 | 0.80 | 0.99 | 1.36 | 0.96 | 1.50 | 0.23 | 0.72 |
| 79 | Xigaze | 0.88 | 0.62 | 2.16 | 1.06 | 2.03 | 1.13 | 1.15 | 2.22 | 0.88 | 3.35 | 0.81 | 1.01 | 1.39 | 1.03 | 1.54 | 0.23 | 0.80 |
| 80 | Xigaze | 0.93 | 0.68 | 2.27 | 1.13 | 2.13 | 1.19 | 1.20 | 2.36 | 0.95 | 3.69 | 0.86 | 1.07 | 1.47 | 0.91 | 1.62 | 0.21 | 0.82 |
| 81 | Xigaze | 0.84 | 0.61 | 2.06 | 1.01 | 1.92 | 1.07 | 1.07 | 2.11 | 0.85 | 3.32 | 0.77 | 0.93 | 1.31 | 0.84 | 1.46 | 0.23 | 0.72 |
| 82 | Xigaze | 0.84 | 0.57 | 1.98 | 0.98 | 1.87 | 1.04 | 1.03 | 2.03 | 0.82 | 3.13 | 0.77 | 0.95 | 1.29 | 0.73 | 1.44 | 0.23 | 0.73 |
| 83 | Xigaze | 0.86 | 0.41 | 2.08 | 1.03 | 1.98 | 1.09 | 1.06 | 2.13 | 0.85 | 3.44 | 0.80 | 0.97 | 1.33 | 0.65 | 1.50 | 0.24 | 0.73 |
| 84 | Xigaze | 0.87 | 0.38 | 2.11 | 1.03 | 1.98 | 1.10 | 1.09 | 2.10 | 0.85 | 3.33 | 0.81 | 1.00 | 1.35 | 0.78 | 1.49 | 0.25 | 0.71 |
| 85 | Xigaze | 0.79 | 0.48 | 1.89 | 0.97 | 1.78 | 0.99 | 1.03 | 1.95 | 0.85 | 3.15 | 0.77 | 0.97 | 1.27 | 0.62 | 1.41 | 0.28 | 0.67 |
| 86 | Xigaze | 0.76 | 0.26 | 1.84 | 0.91 | 1.74 | 0.97 | 0.97 | 1.85 | 0.75 | 2.90 | 0.72 | 0.88 | 1.20 | 0.81 | 1.30 | 0.22 | 0.61 |
| 87 | Xigaze | 0.83 | 0.36 | 1.99 | 0.99 | 1.88 | 1.04 | 1.02 | 2.02 | 0.84 | 3.24 | 0.80 | 0.98 | 1.27 | 0.65 | 1.45 | 0.26 | 0.71 |
| 88 | Xigaze | 0.88 | 0.58 | 2.10 | 1.04 | 1.97 | 1.11 | 1.13 | 2.15 | 0.87 | 3.32 | 0.83 | 1.00 | 1.35 | 0.85 | 1.50 | 0.27 | 0.75 |
| 89 | Xigaze | 0.86 | 0.61 | 2.08 | 1.03 | 1.94 | 1.07 | 1.11 | 2.14 | 0.85 | 3.23 | 0.82 | 1.01 | 1.35 | 0.94 | 1.49 | 0.25 | 0.72 |

**Table S2.** Composition and concentrations of seventeen amino acids in Tibetan chicken from different cities. Values are means±SD.

| Amino acids | All cities | Lhasa | Shannan | Linzhi | Xigaze |
|---|---|---|---|---|---|
| Phe (g/100 g) | 0.91±0.10 | 0.96±0.08[a] | 0.85±0.09[b] | 0.97±0.10[a] | 0.86±0.04[b] |
| Met (g/100 g) | 0.57±0.13 | 0.66±0.09[a] | 0.52±0.13[b] | 0.63±0.08[a] | 0.48±0.13[b] |
| Lys (g/100 g) | 2.14±0.22 | 2.24±0.19[a] | 1.98±0.20[b] | 2.27±0.19[a] | 2.07±0.10[b] |
| Thr (g/100 g) | 1.07±0.11 | 1.14±0.10[a] | 1.00±0.10[b] | 1.11±0.09[a] | 1.02±0.05[b] |
| Leu (g/100 g) | 1.98±0.19 | 2.03±0.17[ab] | 1.85±0.19[c] | 2.09±0.18[a] | 1.95±0.10[bc] |
| Ile (g/100 g) | 1.12±0.11 | 1.16±0.10[a] | 1.04±0.11[b] | 1.18±0.10[a] | 1.09±0.06[b] |
| Val (g/100 g) | 1.15±0.13 | 1.22±0.11[a] | 1.05±0.11[b] | 1.24±0.11[a] | 1.09±0.06[b] |
| Asp (g/100 g) | 2.19±0.22 | 2.34±0.20[a] | 2.05±0.23[b] | 2.28±0.19[a] | 2.10±0.11[b] |
| Ser (g/100 g) | 0.90±0.09 | 0.96±0.09[a] | 0.83±0.09[b] | 0.94±0.07[a] | 0.85±0.04[b] |
| Glu (g/100 g) | 3.45±0.36 | 3.63±0.28[a] | 3.19±0.33[b] | 3.66±0.31[a] | 3.31±0.17[b] |
| Pro (g/100 g) | 0.83±0.08 | 0.86±0.08[a] | 0.78±0.08[b] | 0.88±0.06[a] | 0.80±0.03[b] |
| Gly (g/100 g) | 1.02±0.10 | 1.09±0.11[a] | 0.97±0.10[b] | 1.05±0.08[a] | 0.99±0.04[b] |
| Ala (g/100 g) | 1.37±0.12 | 1.43±0.12[a] | 1.31±0.13[c] | 1.40±0.11[ab] | 1.34±0.06[bc] |
| His (g/100 g) | 0.84±0.13 | 0.97±0.12[a] | 0.78±0.10[b] | 0.83±0.10[b] | 0.81±0.12[b] |
| Arg (g/100 g) | 1.52±0.15 | 1.60±0.14[a] | 1.41±0.14[b] | 1.62±0.12[a] | 1.47±0.07[b] |
| Cys (g/100 g) | 0.23±0.03 | 0.22±0.04[c] | 0.24±0.03[ab] | 0.23±0.02[bc] | 0.25±0.02[a] |
| Tyr (g/100 g) | 0.76±0.11 | 0.80±0.08[b] | 0.66±0.09[d] | 0.86±0.07[a] | 0.72±0.05[c] |
| EAA (g/100 g) | 8.94±0.94 | 9.40±0.82[a] | 8.30±0.90[b] | 9.50±0.76[a] | 8.55±0.46[b] |
| FAA (g/100 g) | 11.43±1.13 | 12.07±0.99[a] | 10.65±1.11[b] | 12.05±0.89[a] | 10.97±0.53[b] |
| TAA (g/100 g) | 22.06±2.22 | 23.28±1.99[a] | 20.53±2.16[b] | 23.26±1.77[a] | 21.20±1.07[b] |

Note: the letters without common are significantly different ($p < 0.05$). FAA: flavor amino acids; EAA: essential amino acids; TAA: total amino acids.

**Table S3.** Comparision of amino acids with the amino acid pattern profile recommended by the World Health Organization and the Food and Agriculture Organization of the United Nations (WHO/FAO).

| Amino acids | Recommended value by WHO/FAO | Lhasa | Shannan | Linzhi | Xigaze |
|---|---|---|---|---|---|
| (Phe+Tyr)/TAA (%) | 6.00 | 7.57±0.14 | 7.37±0.14 | 7.86±0.29 | 7.44±0.10 |
| (Met+Cys)/TAA (%) | 3.50 | 3.73±0.27 | 3.67±0.50 | 3.70±0.37 | 3.42±0.52 |
| Lys/TAA (%) | 5.50 | 9.62±0.14 | 9.67±0.14 | 9.77±0.18 | 9.75±0.11 |
| Thr/TAA (%) | 4.00 | 4.88±0.04 | 4.86±0.06 | 4.79±0.07 | 4.82±0.05 |
| Leu/TAA (%) | 7.00 | 8.71±0.12 | 9.01±0.12 | 8.97±0.16 | 9.19±0.13 |
| Ile/TAA (%) | 4.00 | 4.98±0.13 | 5.09±0.08 | 5.08±0.08 | 5.13±0.08 |
| Val/TAA (%) | 5.00 | 5.23±0.10 | 5.11±0.08 | 5.33±0.11 | 5.13±0.07 |
| EAA/TAA (%) | 40.00 | 40.37±0.52 | 40.40±0.33 | 40.83±0.36 | 40.31±0.29 |
| FAA/TAA (%) | — | 51.84±0.44 | 51.92±0.40 | 51.82±0.67 | 51.75±0.50 |

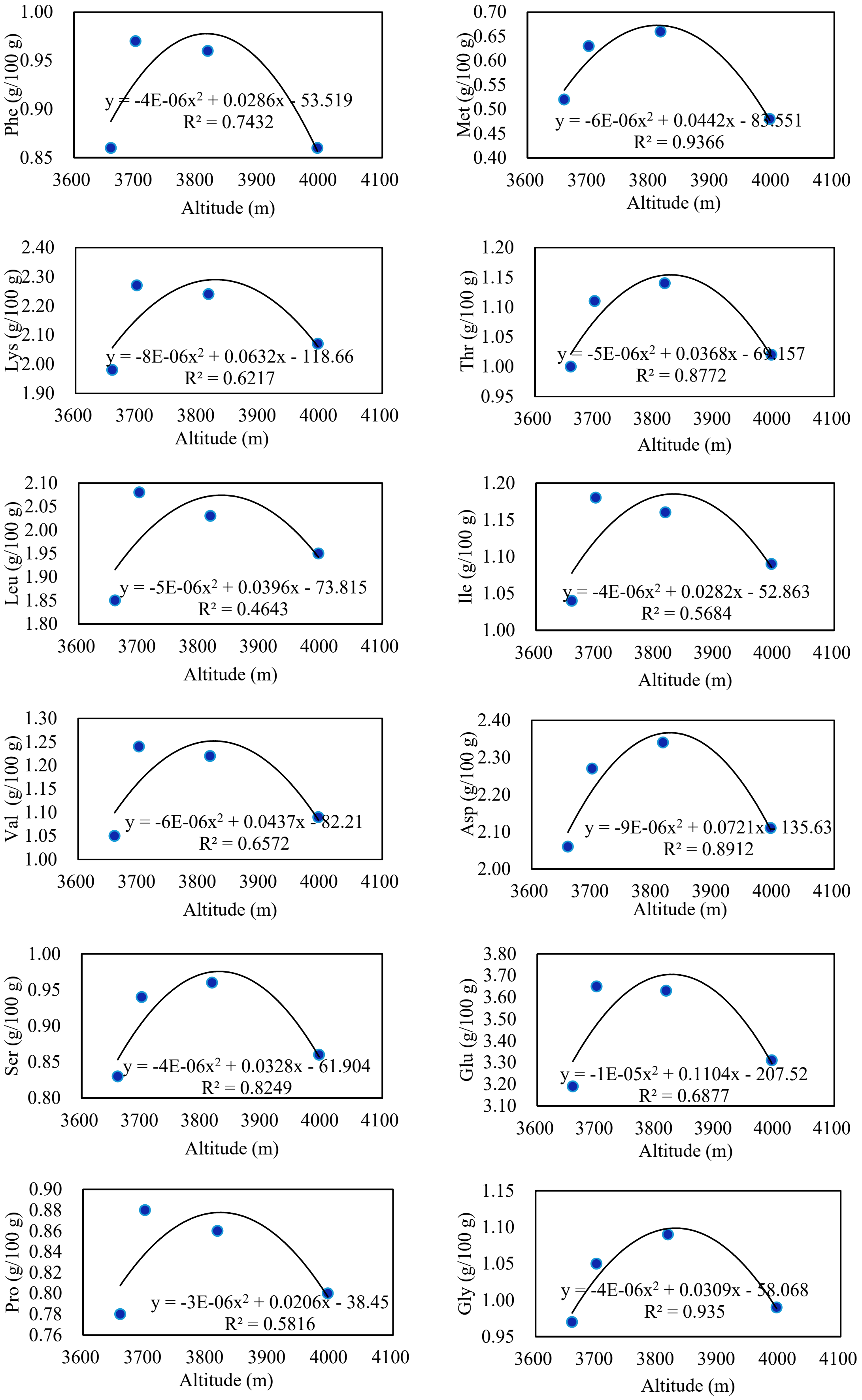

Phe (g/100 g)
y = -4E-06x² + 0.0286x - 53.519
R² = 0.7432
Altitude (m)
Met (g/100 g)
y = -6E-06x² + 0.0442x - 83.551
R² = 0.9366
Altitude (m)
Lys (g/100 g)
y = -8E-06x² + 0.0632x - 118.66
R² = 0.6217
Altitude (m)
Thr (g/100 g)
y = -5E-06x² + 0.0368x - 69.157
R² = 0.8772
Altitude (m)
Leu (g/100 g)
y = -5E-06x² + 0.0396x - 73.815
R² = 0.4643
Altitude (m)
Ile (g/100 g)
y = -4E-06x² + 0.0282x - 52.863
R² = 0.5684
Altitude (m)
Val (g/100 g)
y = -6E-06x² + 0.0437x - 82.21
R² = 0.6572
Altitude (m)
Asp (g/100 g)
y = -9E-06x² + 0.0721x - 135.63
R² = 0.8912
Altitude (m)
Ser (g/100 g)
y = -4E-06x² + 0.0328x - 61.904
R² = 0.8249
Altitude (m)
Glu (g/100 g)
y = -1E-05x² + 0.1104x - 207.52
R² = 0.6877
Altitude (m)
Pro (g/100 g)
y = -3E-06x² + 0.0206x - 38.45
R² = 0.5816
Altitude (m)
Gly (g/100 g)
y = -4E-06x² + 0.0309x - 58.068
R² = 0.935
Altitude (m)

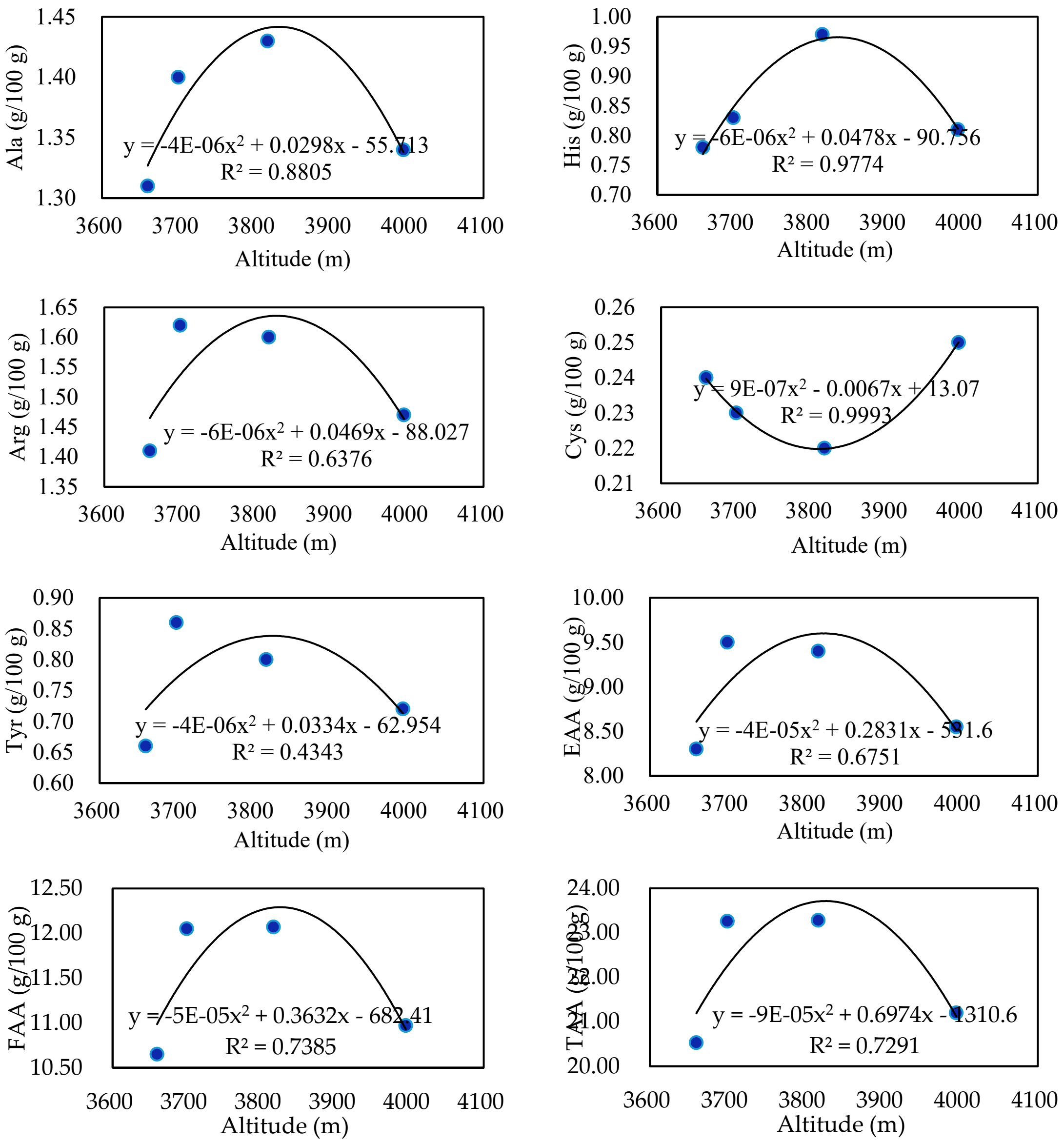


**Figure S1.** Relationship between means amino acids concentration and altitude of different cities
